# Interpreters' experiences of participating in an introduction course in the healthcare sector. An ethnographic field observation study

**DOI:** 10.1002/nop2.736

**Published:** 2020-12-09

**Authors:** Dorthe S. Nielsen, Leila S. Abdulkadir, Charlotte Rehling, Morten Sodemann

**Affiliations:** ^1^ Migrant Health Clinic Odense University Hospital Odense Denmark; ^2^ Center for Global Health University of Southern Denmark Odense Denmark; ^3^ Health Sciences Research Center University Colle of Lillebaelt Odense Denmark

**Keywords:** cultural competence, culture, education, health care, interpreter, language barriers

## Abstract

**Aim:**

We aimed to explore how interpreters experienced being part of a special designed health introduction course and to obtain their perspectives on the learning process. The overall aim was to improve health care to patients with language barriers.

**Design:**

With a hermeneutic phenomenological approach, we used participant observations as a method for collecting data.

**Method:**

The teaching methods used were case‐based learning, role‐play and active involvement including dialogs, discussions and critical reflections. The interpreters were divided into teams of 10–15 interpreters. Over the duration of 6 weeks, teaching sessions were conducted, with 3 hr/week planned.

**Results:**

The findings emphasize the importance of providing the interpreters with education, supervision and a work environment where they can confidentially share and get competent feedback on their experiences and linguistic skills, in order to ensure patients get the correct information in their native language.

## 1 INTRODUCTION

Language barriers are associated with impaired patient safety, misunderstood or incorrect diagnosis and treatment (Flores, 2005; Flores et al., 2003), low patient satisfaction (Drewniak et al., 2017) and longer duration of hospital stay (Hudelson et al., 2014). Several studies have demonstrated that interpreters' knowledge, language and interpretations skills vary, with serious consequences for the patients' safety (Bischoff & Denhaerynck, 2010; Bischoff & Hudelson, 2010; van Rosse et al., 2016).

## 2 BACKGROUND

It is evident that interpreters are important, resourceful persons (Basu et al., 2017; Flores, 2005) with a significant power to bridge patient's personal experiences and doctors' professional knowledge (Hadziabdic & Hjelm, 2013). Interpreters' competencies and working conditions have an impact on patient's experiences of the interpretation situation (Gray et al., 2011; Schapira et al., 2008). Moreover, this has an impact on patients and health professional impression of the given information and communication (Hadziabdic & Hjelm, 2013; Wiking et al., 2009).

Working in the healthcare sector as interpreter is demanding, because the interpreter is solely responsible for an effortless communication between the patient and the healthcare professionals (Bischoff & Hudelson, 2010; Hadziabdic & Hjelm, 2013). Language defines us as persons with preferences, experiences and emotions, and therefore, it is consequential to obtain a form of agreement, on the importance of and values attached to central topics, such as suffering, illness, pain, sorrow, worrying, palliation, care or treatment (Kleinman et al., 1978). Other factors that are involved in the quality of the interpretation are the education of the interpreter, amount of experience (Itani et al., 2017) and the education of the health professionals, but also the environments the interpretation are held have importance. Studies have shown that some interpreters lack knowledge about the healthcare sector, a lack of knowledge about medical terms and some lack of fundamental skills in ethics and interpretation techniques (Flores et al., 2003; Hadziabdic et al., 2014, 2015; Hadziabdic & Hjelm, 2013).

In Denmark, the interpreters are mostly bilingual persons, without formal training apart from the informal training they acquire through experience (Jensen et al., 2010). Often, the bureaus that employ the interpreters are responsible for organizing training. However, some companies have no interests in properly educating their interpreters, due to the extra costs. There is no law demanding interpreters have certain levels of education or language skills; however, education for interpreters is strongly recommended by both government, researchers (Hansen & Nielsen, 2012; Nielsen, 2016) and the Danish institute of Human Rights (Slot, 2014).

In 2017, there was initiated a project by the Region of Southern Denmark, with the aim of creating a more competent group of healthcare interpreters. The region decided that its interpreters should become more qualified through a formal training programme. Through the programme, the expectation was that the interpreters obtained a basic level of knowledge and competency about interpretation techniques, ethics and communication skills, and the most used medical vocabulary, though the aim of the course initially was not to increase overall language competencies. The knowledge about interpreters' perspectives on education and training programmes is sparse.

## 3 AIM

The aim of this project was to explore how the interpreters experienced being part of this special designed health introduction course and to obtain their perspectives on the learning process.

### 3.1 Research question

- How does interpreters experience being on a health introduction course?
- How do interpreters interact with health professionals and other interpreters in the learning process?

## 4 METHODS

### 4.1 Design

With a hermeneutic phenomenological approach (Laverty, 2003), we used participant observations as a method (Spradley, 2016) for collecting data. Participant observation refers to the research methods in ethnography as a study of people and cultures, where the researcher is studying one or more people by participating in their activities (Wray et al., 2007).

### 4.2 The introduction course

The introduction course for the interpreters used the pedagogical principles about active participation and situated learning (Lave & Wenger, 1991). The methods used were case‐based learning, role‐play and active involvement including dialogs, discussions and critical reflections. The interpreters were divided into teams of 10–15 interpreters. Over the duration of 6 weeks, teaching sessions were conducted; with 3 hr/week planned, the content of all lessons are outlined in Table 1. After six months, a two‐hour follow‐up seminar with supervision was planned.

**Table 1:** Content of the six lessons

Lesson 1	Lesson 2	Lesson 3	Lesson 4	Lesson 5	Lesson 6
The Danish healthcare system	Video interpretation, telephone interpretation, onsite interpretation	Basic anatomy and physiology	Illness and cultural differences	Taboos and mental illness	The difficult conversation
Being professional	Focus on the interpreter's perspective: challenges and barriers	Most common diseases	Health perception	Post‐traumatic stress	Summing up
Code of ethics for interpreters	Exercises and visual facilities	Most common treatments	Translation of difficult understanding of treatment and diseases	How to take care of yourself?	Evaluation

The introduction course was developed and tested through a pilot study, with involvement from a group of 10 experienced interpreters, with special competencies in the healthcare sector. Furthermore, patients and healthcare professionals from a specialized migrant health clinic took an active part in developing the teaching programme. Teachers were all experts in Migrant Health, and all were experienced in working with interpreters and patients with limited Danish proficiency.

### 4.3 Participants

A snowball sampling method was applied to recruit participants to the study; they were recruited with the help from other interpreters and gatekeepers working in the healthcare sector. Furthermore, social media, for example Facebook, was used as a platform to spread the message about the introduction programme and for inclusion of voluntary participants in the study.

Over a period of one year, a total of 100 interpreters signed up for the programme. Of these, 87 participants completed the entire course. The gender distribution among the participants was 62% women and 38% men. Almost half of the participants spoke Arabic. Other languages covered were Albanian, Bosnia, Croatia, English, Farsi, Greenlandic, Kurdish, Lithuanian, Polish, Turkish, Russian, Persian, Serbian, Somali and Vietnamese. The length of experience varied among the interpreters; 17 interpreters had more than 15 years of experience, and only five had <1 year of experience. All interpreters had more or less experiences with interpreting in the healthcare sector, which made it possible to have interactive discussions and cases building on the interpreters' previous experiences. Very few participants had courses or education in interpretation in general.

### 4.4 Participant observation

Participant observations aim at looking and describing the cultural differences participants use to organize and to interpret their experiences. This is a constant iterative process. Ethnography work is to observe a social situation with actors and activities. According to James Spradley (Spradley, 2016), a social situation can be identified by three elements: *A place*, in our study the introduction course, *Actors*, which in the current study were the interpreters and teachers and *Activities,* which was the interaction between the actors. Spradley's framework consisting of nine domains was used in the current study, to focus the observations on the aim of the study (Spradley, 2016), further described in Table 2.

**Table 2:** Overview of Spradley's framework consisting of nine domains (Spradley, 2016)

The Nine Domains:		
1.	Space	The physical setting, such as rooms, places and locations
2.	Actors	The people involved in the study.
3.	Activities	The activities conducted by the actors.
4.	Objects	The physical elements involved in the activities and space, used by the actors.
5.	Acts	The individual actions taken by actors.
6.	Events	Context of the acts, actors and space, such as a meeting or a dinner.
7.	Time	The sequence of events from beginning to end.
8.	Goals	What the actors seek to accomplish in their acts.
9.	Feelings	What emotions the actors express in the events.

All authors in turn wrote down field notes, but one research assistant, with LSA, attended all lessons and her observations was recorded in quick summaries during discussions and after teaching. The focus was on the spoken words and the reactions towards the subjects discussed. Furthermore, relations, interactions and feelings among the interpreters and between the interpreters and teachers were observed and recorded. We provided a thick description and a robust data with a wide possible range of information through the detailed and accurate descriptions of the observations in the classroom. Furthermore, we conducted informal interviews with participants to get more in‐depth information on the observed themes arising during the observations.

### 4.5 Analysis

All field notes were analysed, using a cultural thematic theme analysis influenced by Spradley (Spradley, 2016). The analysis consisted of four steps. First, the field notes were systematically read and reviewed. Second, condensated text related to the nine domains (Table 2) was identified throughout the material. The third step of the analysis consisted of a systematic search for similarities and contrasts in the identified domains and the empirical data reduced to decontextualized selection of themes, sorted into a few subgroups. The fourth step of the analysis consisted of synthesizing, moving from field notes to identified domains and themes. This iterative process ensured that the validation of identified cultural themes based the original context and transcripts.

Rigour was archived by constantly checking observations and interpretations with the participants from the course; this included involving the teachers in the analytical process. Through reflexivity and bracketing, the research team was always on guard of own biases, assumptions, beliefs and presuppositions that they could bring to the analysis and findings.

## 5 ETHICS

All participants were introduced to the study, both orally and in writing and all gave written consent to participate in the study. The teaching of and participation in the course was on a voluntary basis and free of any expenses and all participants were informed that they could withdraw at any time. The study was approved by the [BLINDED] (No17/45557), and all data were handled and stored in accordance with Danish law about data protection and GDPR rules. Participants were informed about confidentiality and that they would be unrecognizable in the quotes. Therefore, presented quotes are without any ID or group number.

## 6 FINDINGS

In all classes, there was a very trusting and acknowledging environment and most participants shared their experiences openly. The teachers established an open space for discussions, but simultaneously encouraged participants to uphold confidentiality. Participants provided the class with cake or other kind of snacks and in turns and the teachers prepared coffee and tea. Because of the intimate and trusting environment of the classroom, the observers' roles shifted between being passive and as being an active part of the group.

Based on the analysis, we identified three universal cultural themes:

- Being caught in translation—challenges connected to translation
- Being seen as a person—going from being an interpreter machine to being an important colleague
- Being guided and supervised—acquiring competencies, knowledge and awareness

### 6.1 Caught in translation—challenges connected to being an interpreter

Challenges related to translating words or health conditions were discussed in all sessions. Many of the participants described uncertainty on how to translate specific health terms, or rare health conditions, into a foreign language and from a foreign language into Danish'. Some participants revealed that for them was “osteoarthritis” difficult to translate into Arabic; a Russian interpreter told she had challenges with translating “a crick in one's neck” and for others, the concept of “stress,” was simply being busy. There were two strategies evolving about how to tackle these translating challenges; one strategy was guessing the meaning behind the term and not revealing insecurity or lack of competencies, from a fear of losing one's job, or getting complaints from the healthcare worker whom they were translating for:

I didn't know how to translate a word and I asked the doctor about the literal meaning of the word – he was humiliating me and asked me how long I had been an interpreter.

Another strategy to handle translation challenges expressed by participants was to moderate the translation with respect for the individual patients' cultural beliefs. Hence, cultural beliefs and meaning became important topics in all courses of discussion. Diseases, like cancer and dementia, were in many languages difficult to translate, due to cultural beliefs or the lack of words in the native language. Dementia in Arabic was explained as being the same as “being stupid as a sheep” and no one wanted to translate that to patients:

I never translate cancer to cancer. I always wait until the patient have showed me what terms she or he prefers, many patients do not say the word cancer because of superstition, or because of a fear of dying.

The aim was to try to be sensitive in the delivery of the diagnoses to the patient, and trying to make the doctor or nurse understand the patients' emotional and cultural position. The different strategies and positions were discussed in the classrooms and the teachers were working with the participants' sense of self, with regard to becoming more aware of the responsibilities connected to being an interpreter. A consensus about the importance of being open about difficulties with translation was obtained in all classrooms:

I sometimes experience patients who don't understand any of the doctor's information. From now on I will tell the doctor and stop the conversation [for clarification].

Being involved at a personal level, when translating, was one of the challenges experienced and shared by the interpreters during the course. Some participants had experienced patients asking them about their personal views on different topics, receiving calls late at night, inquiring about personal assistance, or being contacted by relatives asking for a summary of their relatives' conversation or treatment at the hospital. In extreme cases, participants had experienced being threatened by patients or relatives; “The patient said to me; I will come after you if you don't help me” and another participant described a situation where a patient had blamed him for not translating correctly:

The patient got so angry at me after the consultation and shouted that I had translated his condition and wishes in a wrong way.

Others described that the personal contact to patients could present an ethical dilemma; they felt challenged in situations when the patients desperately needed them as a person, because the healthcare professionals showed no understanding or empathy towards the patient. Situations with patients being externalized, desperate or in emotional despair and healthcare professionals demanding the interpreter stay put and not show any feelings or sympathy were shared in most sessions:

I was interpreting for a depressive patient, she threatened to commit suicide, she was so unhappy – I just had to stay with her, but the doctor became angry because I stayed and my boss became angry, because I had to cancel my next appointment – I had no one to discuss or share my experience with.

### 6.2 Being seen as a person—going from being an interpreter machine to being an important colleague and a human being

In most of the groups, there was an agreement among participants that being an interpreter presented with a low social status and lack of respect by others. Many had experienced treatment as an interpreter machine and not respected for their competencies and completed tasks. Participants shared experiences and circumstances of disrespect from doctors and other healthcare workers and secretaries, who had been rude and insulted them in different ways:

As an interpreter, you are not supposed to show your feelings or your emotions – you are just expected to translate and do what the doctor or secretary tells you to do.

Few participants had experienced health professionals' praise or acknowledging them for their work and for interpreting, which for them had high importance and an impact on their sense of self. All groups discussed being seen as a colleague by healthcare professionals, and during the course, it became evident that the participants' self‐image of being an interpreter, changed into being an important, professional and competent colleague for the healthcare professionals:

For the first time in my life, I feel proud about being an interpreter – I am proud of myself and I feel I am needed and people like me and respect me

Some of the participants explained that they paid the price for being an interpreter in their private lives. Being an interpreter made them aware of many people's secrets and vulnerable stories. Most interpreters are a part of a minority group and due to their background; this could entail exclusion from social celebrations and gatherings in their own social circle:

Being an interpreter makes you lonely in your private life – people don't invite you because you know some of their biggest secrets or some of their painful stories.

Attending the introduction course solidified for many participants, the fact that being an interpreter was also about being a person with feelings and emotions. Most interpreters had oppressed their feelings over a period, sometimes spanning years. They had felt akin to a machine, which for some participants resulted in them having to stop working as interpreters for a time or were emotional stressed and affected by their job.

### 6.3 Being guided and supervised—getting competences, knowledge and awareness

During the course, it became evident that all participants felt a general enthusiasm for continuing professional training and supervision. Some had never attended any course on interpretation; others had participated in several courses. Participants working conditions and opportunities for further education varied, largely dependent on the company and the manager for whom they worked.

The first group of interpreters experienced and described the discussions and supervision, as an important professional opportunity that they lacked in their everyday work. Therefore, all instructors decided to use and involve participants' experiences and cases from their work life in the lessons:

We need more teaching, especially about diseases and treatment, but also on our own reactions to patient stories and their conditions.

Being together with colleagues, sharing knowledge, experiences and feelings were highlighted as important experiences missing in most of the participants work life. One participant expressed the following to his fellow students after the course:

This is the first time I have been sharing experiences with colleagues. I have learned a lot from all of you. Thank you.

Being emotional during interpretation had impact on most of the participants; several had experienced starting to cry or being emotional during an interpretation job. This made them feel unprofessional, because expressing emotions, for some colleagues, had resulted in complaints from healthcare staff. The introduction course presented and discussed, different strategies, with the aim to highlight and support the concept, that interpreters also have feelings:

Now I know, I'm not the only one who has been crying during difficult consultations ‐ and I still consider myself as being professional. That is such a relief to be able to accept that.

An issue shared and discussed in all sessions was the need and wish for pre‐briefing before an interpreting job. All participants expressed a strong wish for the opportunity to be prepared when they got an interpretation task. Not knowing if they were going to interpret for parents that had a dying child, or a person that had cancer, or a cured and healed person was cause for frustrations and uncertainty:

We are never prepared for the context. One time I experienced that, I had to interpret for a family with a dying family member. I had no idea what was going on until I suddenly was in a room among a lot of crying and very unhappy family members – I would have declined the job if I had known […] I had just lost my own mother.

Not having the opportunity to be prepared for what they were going to interpret and to whom, for many participants caused a lot of worries and stress, but sharing the experiences confirmed to the individual participant that they were not alone with their feelings.

## 7 DISCUSSION

The findings of this study highlight that participating in the current introduction course had significance for most participants. The introduction course supported the interpreter's ability to see them self as important colleagues to healthcare professionals. Furthermore, they learned they were not alone with their feelings of frustrations and emotional responses, and most importantly, they learned from each other new ways of seeing themselves and understanding their role as interpreters. Similar issues has been described by Schapira et al. (2008) who furthermore put focus on the interpreters working conditions, as a prerequisite for developing competencies, awareness and sensitivity among interpreters. They highlighted the importance of establishing collaborative practices with interpreters (Schapira et al., 2008). Our results underline that experiences from the participants' work life, for many had an impact on their private and social life and on their sense of self. Being called up outside of working hours, being treated as an interpreter machine, being excluded from ones social network, had in many ways existential consequences for the interpreters, making them vulnerable and making them rethink their professional identity as interpreters.

According to the interpreters participating in our study, following training participants were important: (a) knowledge about how and where to get support, (b) supervision in difficult interpreting situations; and (c) knowledge and competencies on how to translate vulnerable diagnoses and sensitive patient situations and reactions. Another Danish study underlined the importance of all interpreters to undergo testing of their linguistic skills, to be able to work professionally as healthcare interpreters (Itani et al., 2017). However, linguistic skills alone are not enough. It is not just a question of whether the interpreter can understand the doctor's medical terms and expressions of cancer, stress or dementia (Estrada & Messias, 2018). The interpreter must be able to find the best everyday description, in the patient's native language. Our study demonstrated that interpreters need supervision and a work environment where they can share knowledge and experiences, and getting competent feedback. This is a prerequisite for evolving professional competencies, pride and self‐respect.

In the current study, some participants had experienced scolding when being emotional, or when asking for advice, or when asking about the meaning of a particular medical term. This illustrates that healthcare professionals also need training on how to communicate with interpreters and how to be culturally competent. When the interpreters' are scolded and disrespected, they are underused in regards to their potential and resources (Tiselius et al., 2020). Several studies have demonstrated that serious misunderstandings can lead to mistreatment, or threat of patient safety, when healthcare professionals are not collaborating with professional interpreters (Drewniak et al., 2017; Flores et al., 2003; Hadziabdic et al., 2014). Krystallidou et al. have further demonstrated the importance of both health professionals and interpreters alike, acquire skills on how to collaborate and conduct empathic communication with patients (Krystallidou et al., 2019; Theys et al., 2020). This emphasizes the need for more courses in communication and in relational and cultural skills, aimed at both interpreters and healthcare professionals.

## 8 LIMITATIONS

Using participant observations made it possible to understand the participating interpreters' experiences of acquiring knowledge and new skills at the introduction course. However, when using participant observations as a tool for data collection, it presents with its own limitations. The limitations are associated with the person performing the observations, having access to different actors, settings and pools of knowledge. Participant observation was in our study conducted by researchers participating in most classes. The observations could potentially be biased and influenced by gender, sexuality, ethnicity, class and theoretical approach and thereby affect the observations, written field notes, analysis and interpretation of data (Wray et al., 2007). However, using Spradley's nine domains and thematic analysis (Spradley, 2016) helped the research team to keep focus on the aim of the study and on the actions, interactions and relations being observed in the classroom.

## 9 CONCLUSION AND IMPLICATION FOR PRACTICE

The current study emphasizes the importance of providing the interpreters with education, supervision and a work environment where they can confidentially share and get competent feedback on their experiences and linguistic skills, to ensure patients get the correct information in their native language. Interpreters play a very important role in securing the right information between patients and healthcare professionals, and a good relationship and trust between patients and the healthcare sector. Hence, the importance of training and supervision should not be ignored or underestimated.

Based on the current study, Region of Southern Denmark has already started to employ interpreters at a regional hospital, providing them with training and further education. Structured education and training for interpreters are both needed and sought after by interpreters, patients and healthcare professionals.

## CONFLICT OF INTEREST

We have no conflict to declare.

## AUTHOR CONTRIBUTIONS

DN, MS: Designing the project. DN, LSA, CR, MS: Data collection. DN, MS, LSA, CR: Data analysis. DN, MS, LSA, CR: Draft of paper. DN: Complete the paper.

## ETHICAL STATEMENT

This material is the authors' own original work, which has not been previously published elsewhere. All authors have been personally and actively involved in substantial work leading to the paper, and will take responsibility for its content.

## Data Availability

Due to the nature of this research, participants of this study did not agree for their data to be shared publicly, so supporting data are not available.
